# Familial Mediterranean Fever in older children

**DOI:** 10.1186/1546-0096-12-S1-P254

**Published:** 2014-09-17

**Authors:** Semanur Özdel, Zeynep Birsin Özçakar, Seda Sahin, Mesiha Ekim, Atilla Elhan, Fatos Yalcinkaya

**Affiliations:** 1 Pediatric Rheumatology, Ankara University, Ankara, Turkey; 2 Pediatrics, Ankara University, Ankara, Turkey; 3 Biostatistics, Ankara University, Ankara, Turkey

## Introduction

Familial Mediterranean Fever (FMF) is an autosomal recessive disease, characterised by recurrent, self limited attacks of fever with serositis.

## Objectives

The aim of this study was to compare the demographic, clinical and genetic features of FMF patients who had late onset disease to those with earlier onset during childhood period.

## Methods

Files of patients who had been seen in our department (during routine follow-up visits) between January 2013 and January 2014 were retrospectively evaluated. Patients were divided into two groups according to age of disease onset (Group I: ≤8 years of age; Group II: >8 years of age).

## Results

The study group comprised 317 FMF patients (170 females, 147 males) with a mean age of 12.2 ± 5.7 years. There were 267 patients (84.3%) in group I and 50 patients (15.7%) in Group II. Median attack frequency before colchicine therapy was 24/year in Group I and 12/year in Group II (p<0,05). Although the frequency of majority of the clinical features did not differ between the groups, fever was seen less frequently in Group II patients (p=0,003). M694V homozygosity was also less frequently detected in group II patients (p=0.022). Median disesase severity scores and final colchicine dosages were lower in Group II (p<0,001; p=0,003). Median delay in diagnosis was 24 months in Group I and 12 months in Group II (p=0,002).

## Conclusion

Only a small number of FMF patients had disease onset at older ages in childhood period. It seems that FMF patients with late onset disease have milder illness. However, more readily expression of their clinical findings in older ages yields earlier diagnosis in this group.

## Disclosure of interest

None declared.

